# Quantitative Genetics of Migration-Related Traits in Rainbow and Steelhead Trout

**DOI:** 10.1534/g3.114.016469

**Published:** 2015-03-16

**Authors:** Benjamin C. Hecht, Jeffrey J. Hard, Frank P. Thrower, Krista M. Nichols

**Affiliations:** * Department of Biological Sciences, Purdue University, West Lafayette, Indiana 47907; † Department of Forestry and Natural Resources, Purdue University, West Lafayette, Indiana 47907; ‡ Hagerman Fish Culture Experiment Station, Aquaculture Research Institute, University of Idaho, and Fishery Science Department, Columbia River Inter-Tribal Fish Commission, Hagerman, Idaho 83332; § Conservation Biology Division, Northwest Fisheries Science Center, National Marine Fisheries Service, National Oceanic and Atmospheric Administration, Seattle, Washington 98112; ** Ted Stevens Marine Research Institute, Alaska Fisheries Science Center, National Marine Fisheries Service, National Oceanic and Atmospheric Administration, Juneau, Alaska 99801

**Keywords:** anadromy, animal model, genetic correlation, heritability, steelhead trout

## Abstract

Rainbow trout (*Oncorhynchus mykiss*) exhibit remarkable life history diversity throughout their native range, and among the most evident is variation in migratory propensity. Although some populations and ecotypes will remain resident in freshwater habitats throughout their life history, others have the ability to undertake tremendous marine migrations. Those that migrate undergo a suite of behavioral, morphological, and physiological adaptations in a process called smoltification. We describe a quantitative genetic analysis of 22 growth, size, and morphological traits in addition to overall life history classification (resident or migrant) over the temporal process of smoltification in a large multi-generation experimental pedigree (n = 16,139) of migratory and resident rainbow trout derived from a wild population, which naturally segregates for migratory propensity. We identify significant additive genetic variance and covariance among the suite of traits that make up a component of the migratory syndrome in this species. Additionally, we identify high heritability estimates for the life history classifications and observe a strong negative genetic correlation between the migratory and resident life history trajectories. Given the large heritability estimates of all of the traits that segregate between migratory and resident rainbow trout, we conclude that these traits can respond to selection. However, given the high degree of genetic correlation between these traits, they do not evolve in isolation, but rather as a suite of coordinated characters in a predictable manner.

Migration is a complex life history composed of physiological, morphological, and behavioral traits working in synchrony with environmental cues to move animals over great distances in what has been termed a migratory syndrome (Dingle 2006; Dingle and Drake 2007; Pulido 2007). Traits involved in migratory syndromes have been shown to be heritable (Roff and Fairbairn 2007; Teplitsky *et al.* 2011) and, as such, migratory syndromes could respond to natural selection (Dingle 2006) and are potentially adaptive (Pulido 2007). However, traits do not evolve in complete isolation, but in a coordinated fashion with other characters. The evolutionary potential of traits thus relies not only on the amount of additive genetic variation in single traits but also on genetic covariance among traits. As evolution advances along the path with the least genetic resistance and/or the greatest genetic variance, genetic correlations, depending on their sign, may affect the rate and direction of the response to natural selection (Falconer and Mackay 1996; Lynch and Walsh 1998) and even generate adaptive constraints (Blows and Hoffmann 2005; Walsh and Blows 2009; Teplitsky *et al.* 2011). An understanding of the evolutionary potential and adaptive significance of migration will require insight into the patterns of variation and covariation among the suite of correlated traits that make up the migratory syndrome, as well as the selective forces acting on them (Roff and Fairbairn 2007).

Morphological characteristics play a critical role in the ability of an individual to successfully migrate. Several studies have identified morphological variation between resident and migratory forms of animals, including variation in wing morphology of birds and insects (Mulvihill and Chandler 1990; Dingle 2006; Roff and Fairbairn 2007; Tarka *et al.* 2010), head morphology associated with differing food habits (Dingle 2006), the streamlined body shape in migratory types compared to resident conspecifics (Webb 1984; Fraser and Bernatchez 2005; Nichols *et al.* 2008), and coloration of birds (Fitzpatrick 1994) and fishes (Hoar 1976) as adaptations to varying habitats and environmental resources experienced by migrants relative to residents.

Although life history and morphological variation are pervasive between all manner of migratory and resident forms of animals, this variation is perhaps best appreciated among the salmonid (salmon, trout, and char) family of fishes, wherein great variation exists in the propensity, timing, duration, and distance of their marine migrations (Quinn and Myers 2005). Two general life history tactics occur within salmonids: a “resident” life history, wherein individuals complete their entire life cycle within their natal freshwater habitat, and a migratory “anadromous” life history where, after a period of juvenile growth and development in freshwater, individuals migrate out to sea to take advantage of productive marine environments before returning to their natal freshwater habitat to spawn (Quinn 2005). Before migrating to sea, salmonids undergo a process called “smoltification,” which involves a cascade of physiological, biochemical, morphological, and behavioral changes triggered by environmental cues to transition freshwater adapted “parr” into marine adapted “smolts” (Hoar 1976; Folmar and Dickhoff 1980). It has been hypothesized that smoltification is really a developmental decision made in individuals that have failed to reach sexual maturity in their freshwater habitat; therefore, sexual maturation in freshwater might preclude a marine migration (Thorpe 1994; Thorpe and Metcalfe 1998).

The morphological changes that take place during the smoltification process in salmonids include changes in body shape, growth rates, body condition, and coloration (Hoar 1976; Folmar and Dickhoff 1980; Beeman *et al.* 1994, 1995; Dickhoff *et al.* 1997), and all are considered components of the migratory syndrome in salmonids. Shape changes include an elongation of the body to a more streamlined profile with noticeable lengthening of the caudal peduncle (Winans and Nishioka 1987; Hard *et al.* 1999). This shape change reduces drag, allowing for sustained long distance swimming (Webb 1984). Smolts also have a lower body condition when compared to non-smolts (Hoar 1976; Folmar and Dickhoff 1980), which is a measure of the relationship between body length and weight (Nash *et al.* 2006), and quantifies the level of “plumpness” in a fish. Additionally, smolts can experience higher levels of growth, especially in body length, in the months leading to outmigration when compared to non-smolts (Folmar and Dickhoff 1980; Dickhoff *et al.* 1997). Perhaps the most striking difference between freshwater adapted parr and marine adapted smolts are differences in body coloration. The metabolic byproducts guanine and hypoxanthine are deposited in the skin and scales of smolts, turning them from dark colored parr adapted for rocky shallow freshwater habitats to highly reflective silver morphs better adapted to pelagic marine environments (Folmar and Dickhoff 1980). This silver reflective color is due to the deposition of guanine and hypoxanthine in the skin (Haner *et al.* 1995) and is highly associated with the overall propensity to migrate (Haner *et al.* 1995; Ando *et al.* 2005).

Some evidence suggests that morphology is plastic in salmonids, whereby body shape changes as a result of environmental influence (Pakkasmaa and Pironen 2001; Imre *et al.* 2002; Peres-Neto *et al.* 2006), but there is also evidence that a substantial proportion of morphological variation between migratory and nonmigratory types is attributed to underlying additive genetic variation (Johnsson *et al.* 1994; Thrower *et al.* 2004a; Thériault *et al.* 2007; Varian and Nichols 2010). Quantitative trait loci (QTL) have been identified for several morphological and growth-related traits in experimental crosses of migratory steelhead and resident rainbow trout (*Oncorhynchus mykiss*) (Nichols *et al.* 2008; Hecht *et al.* 2012). As components of a greater migratory syndrome, these size-, growth-, and morphological-related traits may be under substantial genetic constraint due to genetic correlations between them; indeed, Hard *et al.* (1999) identified large genetic correlations among three-dimensional body shape elements in smolting chinook salmon (*Oncorhynchus tshawytscha*). Moreover, anthropogenic and climate-induced selection may be tremendous driving forces in shaping individual traits and behaviors (Allendorf and Hard 2009; Tuomainen and Candolin 2011). An understanding of how migration-related traits co-vary within the framework of a migratory syndrome can shed light on how migratory species can respond to selection.

In this study, we quantified additive genetic variation and covariation within and between migration-related traits, including body size, growth rates, body coloration, body condition, and body morphology, in addition to binary measures of overall life history status. The specific objectives of this study were to evaluate the following: differences in trait values between life history classes; additive genetic variance for individual morphological and life history trait classification; genetic correlations between traits; and the ability of morphological and growth-related traits to predict overall life history decisions. We use an extensive pedigree of full and half-sib families resulting from pure migratory, pure resident, and intercrosses of steelhead and rainbow trout spanning three generations, originally derived from wild populations of resident rainbow and migratory steelhead trout from southeastern Alaska. Ultimately, this study contributes to our understanding on the quantitative genetic variation underlying a complex life history decision and provides some insight into how evolution might shape the propensity to migrate or remain resident.

## Materials and Methods

### Genetic crosses and samples

The pedigree used in this study is derived from experimental crosses within and between anadromous steelhead and resident rainbow trout from the Sashin Creek watershed in southeastern Alaska (56 degrees 23′ N, 134 degrees 39′ W), which is described extensively elsewhere (Thrower *et al.* 2004a,b). Briefly, in June 1996, anadromous steelhead adults were collected in a weir trap at the mouth of Sashin Creek and the Pacific Ocean as they were returning from their marine migration. Adult resident fish were captured in Sashin Lake upstream and above two barrier waterfalls of the migrant weir trap. Adult resident fish were identified as those that were sexually mature at the time of sampling in freshwater, and because of the barrier waterfalls that prohibit upstream migration could not themselves be misidentified as anadromous migrant returns. These wild captured adults constitute the P_1_ generation and were crossed to produce the second generation (F_1_). Spawning efforts to produce the second generation included the creation of full and half-sib families derived from crosses of presumably unrelated anadromous females to anadromous males (A×A), anadromous females to resident males (A×R), resident females to anadromous males (R×A), and resident females to resident males (R×R). F_1_ offspring were raised in captivity in freshwater as described by Thrower *et al.* (2004a). At 12 months after fertilization, in June 1997, offspring from each family were anesthetized and a passive integrated transponder (PIT) tag was implanted for individual identification. At this same sampling time, in addition to time points 15 months and 24 months after fertilization, morphological measurements were taken for each individual as described below. In this experimental population, most steelhead smolts complete the smoltification process at 24 months after fertilization (Thrower *et al.* 2004a). Thus, at 24 months after fertilization, a qualitative assessment of the overall morphology and coloration of each fish was taken, and a life history classification was assigned to each fish as described in Thrower *et al.* (2004a) and Hecht *et al.*(2012). In this F_1_ generation, all fish that were categorized as residents were reared in freshwater, whereas those fish deemed to be anadromous smolts were transferred to saltwater net pens. In 2004, unrelated mature fish from the captive F_1_ generation were bred to yield F_2_ generation fish. In addition to the crosses outlined above (A×A, A×R, R×A, and R×R), two additional cross-types were made where an A×R steelhead female was crossed to an A×R steelhead male (AR×AR) and an R×A steelhead female was crossed to an R×A steelhead male (RA×RA). Thus, our pedigree consisted of three generations: the original wild-caught P_1_ adults used to produce the initial crosses; the F_1_ progeny derived from the P_1_ and raised in captivity to adulthood; and the F_2_ progeny derived from crosses of unrelated F_1_ parents also raised in captivity. Juvenile phenotypic traits were sampled from F_1_ and F_2_ progeny, as described below.

### Phenotypic traits

To quantify the migratory phenotype in this species, we measured 22 traits, including measures of body size, body condition, growth, morphology, and skin reflectance, all of which have been shown to capture differences between resident and migratory juveniles in this species (Thrower *et al.* 2004a; Nichols *et al.* 2008; Hecht *et al.* 2012). Observations were recorded at three time points during the juvenile growth period, including 12 months after fertilization (“12mo”), 15 months after fertilization (“15mo”), and 24 months after fertilization (“24mo”). Body size and condition were measured at all three time points (12mo, 15mo, and 24mo) and growth rates were measured between the time points (12mo to 15mo and 15mo to 24mo). The remaining phenotypes and a qualitative assessment of life history were measured at 24 months after fertilization, the age and time at which most smolts of this experimental population are expected to smolt (Thrower *et al.* 2004a). At each sampling point fish were anesthetized with MS222 (Argent Laboratories, Redmond, WA). Fork length (the distance from the tip of the snout to the fork in the tail fin, mm), weight (g), and life history categorization (described below) were recorded and photos were taken for later quantification of body morphology and skin reflectance (described below); all phenotypes and abbreviations are summarized in Table 1.

**Table 1:** Description of each phenotypic trait

Trait	Time Point	Description of Trait	Mean	SD	N
LHSmolt	Mo 12	Binary life history trait, smolt = 1, mature/parr = 0, indeterminate = n/a	0.75	0.44	15,021
LHMature	Mo 12	Binary life history trait, mature = 1, parr/indeterminate/smolt = 0	0.13	0.33	15,635
mo12Length	Mo 12	Fork length (mm)	86.47	14.57	16,050
mo12Weight	Mo 12	Weight (g)	7.46	3.9	16,001
mo12Kfact	Mo 12	Body condition factor (K)	1.06	0.1	15,993
mo15Length	Mo 15	Fork length (mm)	168.65	29.2	15,723
mo15Weight	Mo 15	Weight (g)	65.71	35.86	15,702
mo15Kfact	Mo 15	Body condition factor (K)	1.25	0.1	15,692
IGRL1	Mo 12–mo 15	Instantaneous growth rate in length	0.63	0.08	15,708
IGRW1	Mo 12–mo 15	Instantaneous growth rate in weight	2.04	0.25	15,641
mo24Length	Mo 24	Fork length (mm)	211.95	30.16	15,642
mo24Weight	Mo 24	Weight (g)	107.44	49.61	15,641
mo24Kfact	Mo 24	Body condition factor (K)	1.07	0.11	15,634
IGRL2	Mo 24	Instantaneous growth rate in length	0.09	0.03	15,588
IGRW2	Mo 24	Instantaneous growth rate in weight	0.22	0.1	15,571
AvgPix	Mo 24	Skin reflectance (average white pixel intensity)	48.08	30.4	2057
CentroidSize	Mo 24	Centroid size (mm, measure of overall body size)	1813.75	672.65	1985
RelW2	Mo 24	Relative warp 2	−0.16	15.25	1985
RelW3	Mo 24	Relative warp 3	0.03	9.52	1985
RelW4	Mo 24	Relative warp 4	0.12	7.97	1985
RelW5	Mo 24	Relative warp 5	0.05	7.52	1985
RelW6	Mo 24	Relative warp 6	0.06	6.51	1985

Description of each phenotypic trait including an abbreviated trait name used for the phenotype throughout the text, the developmental time point (measured as months (Mo) after fertilization) when the trait was measured, the mean, standard deviation, and the number of observations for each trait.

#### Life history classification:

**Figure 1:**
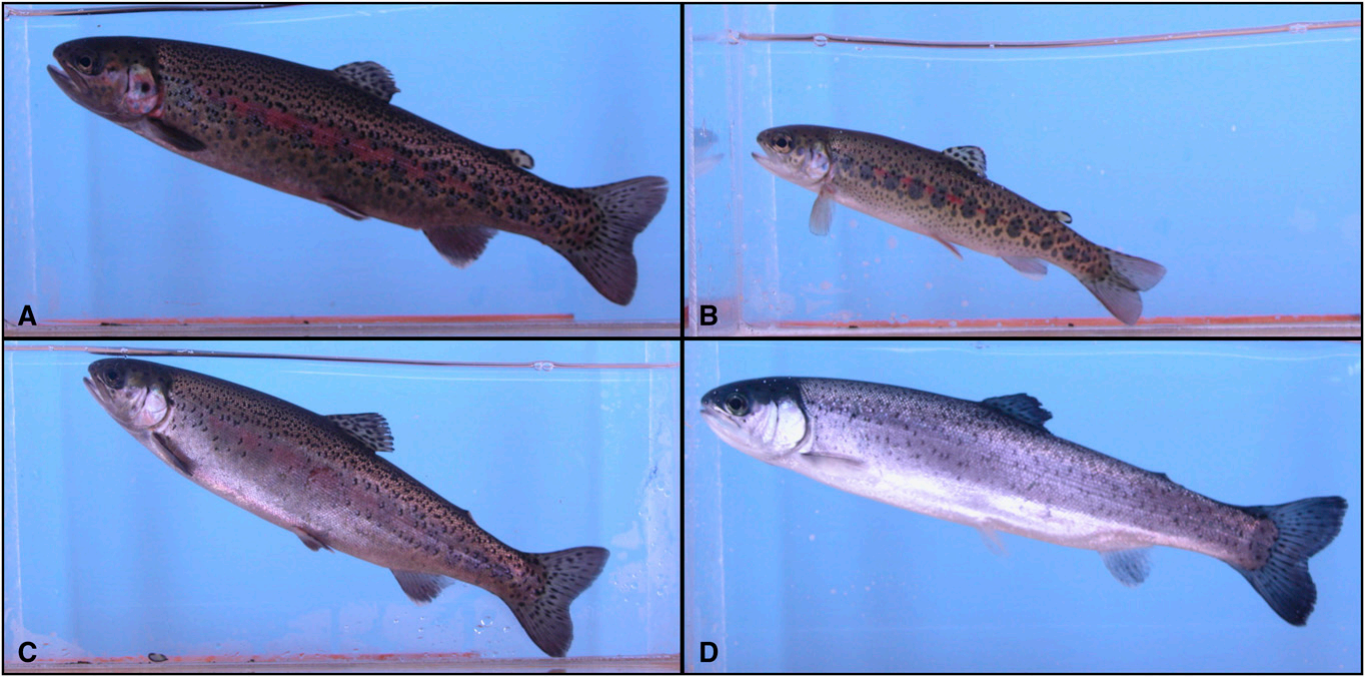
Examples of the four categorical life history classifications in 2-yr-old juvenile rainbow trout from Sashin Creek, Alaska. (A) Precocious “mature” male resident. (B) Immature resident rainbow trout “parr.” (C) An “indeterminate” class. (D) A migratory “smolt.”

Individual life history at age 2 (24 months) was scored as a categorical trait with four levels, the first being precocious mature resident rainbow trout (“mature”) identified by the expression of gametes (sperm or eggs) with the application of gentle pressure to the abdomen. The second classification was for immature resident rainbow trout parr (“parr”), which were identified by an overall dark and colorful body with visible parr marks along the lateral line, reduced fork length relative to the other life history classifications, and failure to express gametes at the time of sampling. Migratory smolts (“smolt”) were the third classification and were identified by having a more streamlined body form (Hoar 1976) with silvery reflective skin, dark back and fins, and a lack of colorful parr marks (Haner *et al.* 1995). Fish with an indeterminate life history (“indeterminate”) were the fourth classification and included those that had failed to complete the smoltification process in their first 2 yr but might otherwise complete the process in future years, or may reach maturation in freshwater and take a resident life history form. Indeterminate individuals had faint but visible parr marks, a lighter colored body, failed to express gametes, and generally were larger in body length than those classified as “parr.” A representative image of each life history classification is provided in Figure 1. Life history classification was decomposed into two binary traits for quantitative genetic analyses: “LHSmolt” and “LHMature.” LHSmolt was scored as a “1” for “smolts” and “0” for “mature” and “parr” classifications, with “indeterminate” fish classified as missing values. LHMature was scored as a “1” for “mature” fish and a “0” for all other life history types.

#### *Body size*, *condition*, *and growth rate:*

Fork length (mm) and body weight (g) were recorded for F_1_ and F_2_ generation fish at 12 (“mo12Length” and “mo12Weight”), 15 (“mo15Length” and “mo15Weight”), and 24 (“mo24Length” and “mo24Weight”) months after fertilization. Body condition factor (K; “Kfact”) is a measure of the contribution of body length to weight, and is a general measure of fish health and condition. Smolting juvenile salmonids have been shown to have a lower body condition when compared to non-smolts (Hoar 1976; Folmar and Dickhoff 1980). Body condition was calculated for each sampling time point (“mo12Kfact,” “mo15Kfact,” and “mo24Kfact”) with the formula K=[(W/L^3^) *100,000], where W is the body weight (g) and L is fork length (mm) at a given time point (Nash *et al.* 2006). Smolting rainbow trout generally experience higher levels of growth in the spring of their second year just prior to their outmigration when compared to non-smolts (Folmar and Dickhoff 1980; Dickhoff *et al.* 1997). To capture this variation, instantaneous growth rates in body length (IGRL) and body weight (IGRW) were calculated across two time periods, from month 12 to month 15 (“IGRL1” and “IGRW1”) and from month 15 to month 24 (“IGRL2” and “IGRW2”) after fertilization. The growth rate was calculated as [ln(L_2_) − ln(L_1_)]/[t_2_ − t_1_] × 100, where L_1_ and L_2_ are lengths (mm) or weights (g) at the first (t_1_, in days after fertilization) and second (t_2_, in days after fertilization) time points in the interval being calculated. Length, weight, body condition factor, and instantaneous growth rates were calculated for both F_1_ and F_2_ generations.

#### Skin reflectance:

**Figure 2:**
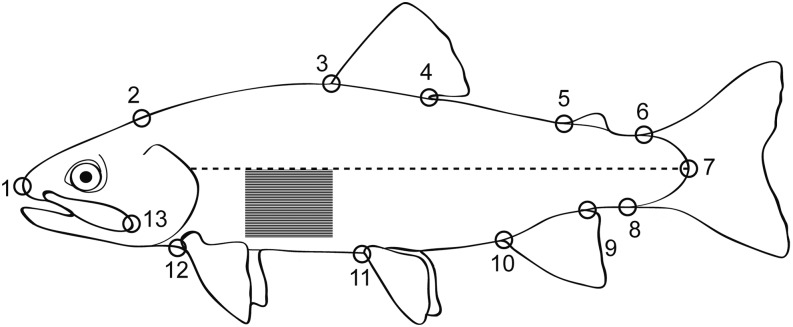
Digitized landmarks (1–13) for thin plate spline analysis of body morphology and area measured for skin reflectance. Skin reflectance was quantified in the shaded region behind the pectoral fin, below the lateral line (dashed line), and before the insertion of the dorsal fin as the average white pixel intensity. Image modified from Nichols *et al.* (2008)

Perhaps the most striking difference between mature and resident parr and marine adapted smolts can be observed in the level of silvering or reflectance in the skin. To quantify skin reflectance, we measured the average white pixel intensity (“AvgPix”) of a defined region behind the pectoral fin, below the lateral line, and anterior to the dorsal fin (Figure 2) in a subset of 2057 F_2_ generation fish including an F_2_ family used for a QTL analysis as described by Hecht *et al.* (2012). This measure was taken at 24 months after fertilization and was calculated from the same digital images used to identify landmarks for the morphometric analysis described below. For additional detailed information regarding the calculation of this measure see Nichols *et al.* (2008) and Hecht *et al.* (2012).

#### Body morphology:

To quantify variation in body shape we used a morphometric analysis of 13 landmarks along the left side of a subset of 2057 F_2_ generation fish including an F_2_ family used for a QTL analysis, as described by Hecht *et al.* (2012). Landmarks were plotted on digital images of each fish following protocols as described previously (Nichols *et al.* 2008; Varian and Nichols 2010) using the software program tpsDig2 (Rohlf 2009). Digitized landmarks from each fish were superimposed to calculate the generalized least-squares Procrustes average or consensus body shape while eliminating differences in body size and after accounting for variation in translation and rotation of images in coordinate space (Rohlf and Slice 1990). Each sample was then analyzed for individual deformation from the consensus shape, and the variation was captured as partial warp scores (Rohlf and Bookstein 2003). Partial warp scores for all individuals were condensed into 22 orthogonal dimensions in a principal components analysis and called relative warps (“RelW1”–“RelW22”). Of the 22 relative warps estimated, only five of the six warps explaining the most variation (“RelW2”–“RelW6”) were used as quantitative metrics for shape variation in this analysis (see *Results* for details). Values for these measures were small, so each relative warp score was multiplied by 1000 for analyses and presentation purposes. In addition to measures of shape variation, this analysis yielded a measure of the overall body size of the fish in the form of a centroid size (“CentroidSize”), which is the square root of the sum of the squared distances from each point to the shape centroid and was calculated at 24 months after fertilization. Figure 2 illustrates the placement of digitized landmarks for relative warps analysis in addition to the region analyzed for skin reflectance described above.

### Statistical analyses

#### Summary statistics:

All statistical analyses were conducted using packages in the R statistical computing environment developed by the R Core Team (2013) unless otherwise noted. Statistical significance was determined at the α = 0.05 level for all tests and comparisons. All traits were investigated for departures of normality and potential outliers were carefully scrutinized. Some samples were not scored for some traits during the collection of phenotypic data, and in these circumstances a missing value was assigned.

To test for significant differences in trait means between life history classes and to determine the overall significance of life history classification on each trait, a single factor ANOVA was conducted. Pairwise *t*-tests between the means for each life history class were estimated using a Tukey-Kramer adjustment for the multiple comparisons. Phenotypic correlations were conducted using a Pearson’s coefficient for quantitative trait correlations or a Spearman’s coefficient for correlations with the binary life history classifications. Discriminant function analyses (DFA) were conducted using the R package MASS (Venables and Ripley 2002) in three ways to determine whether a set of trait variables could accurately predict the life history classification of an individual. In each case, samples missing an observation in a single trait were ineligible for the complete analysis and removed. The first DFA was conducted using both F_1_ and F_2_ samples (n = 15,471) and the traits mo12Length, mo12Weight, mo12Kfact, mo15Length, mo15Weight, mo15Kfact, IGRL1, IGRW1, mo24Length, mo24Weight, mo24Kfact, IGRL2, and IGRW2 to predict the four class categorical life history assignment (mature, parr, indeterminate, and smolt). The second DFA was conducted using the same predictive traits as the first, but it was conducted only on F_2_ samples (n = 8925) to determine whether environmental or year effects contributed to the prediction. A third DFA was conducted using all of the predictive traits from the first two DFAs, but it also included the traits AvgPix, CentroidSize, and RelW2–RelW6 and was only conducted in the F_2_ samples where these additional morphological characters were collected (n = 1882) to test the additional predictive power of the morphological traits.

#### Quantitative genetic analyses:

Variance components used to also estimate trait heritabilities were calculated using a mixed model called an animal model (reviewed in Lynch and Walsh 1998; Kruuk 2004). In an animal model, the phenotype of each individual is broken down to its components of fixed and random sources of variation that can account for nongenetic sources of variation, as well as a random animal effect or breeding value. The breeding value of an individual in the pedigree estimates the individual’s contribution to the phenotype in the population, measured as the deviation of its relatives from the population mean. An animal model takes the basic univariate form:

$$y_{i}=u+a_{i}+e_{i}$$

Where ***y*** is the phenotypic value of individual *i*, *u* is the population mean value, *a* is the breeding value of individual *i*, and ***e*** is a vector of residual errors (reviewed in Kruuk 2004). The total phenotypic variance (V_P_) of each trait can then be described as V_P_ = V_A_ + V_R_, where V_A_ is the additive genetic variance and V_R_ is the residual variance, which consists of environmental variance unaccounted for by additional fixed or random effects, nonadditive genetic variance, and error variance (Falconer and Mackay 1996). The narrow sense heritability (*h^2^*) is the ratio of the additive genetic variance to the total phenotypic variance: *h^2^* = V_A_/V_P_ (Falconer and Mackay 1996). Genetic correlations (*r*_A_) among traits were estimated in the same framework of the animal model using a bivariate analysis of two traits (1 and 2) at one time to generate genetic covariance estimates (${\text{COV}}_{A_{1},A_{2}}$). Genetic correlations (*r*_A_) were calculated as:

$$r_{\text{A}}={\text{COV}}_{A_{1},A_{2}}/\sqrt{V_{A_{1}}V_{A_{2}}}$$

Fixed effects included “generation” (Gen), which captures an overall environmental effect shared among members of the same cohort but not between cohorts, and “cross-type” (xtype), which accounts for both the cross of origin and the rearing environment during the second year of growth after fish were tagged and combined into common raceways based on their cross-type (*i.e.*, all R×R offspring were pooled into one raceway, all A×A offspring were pooled into one raceway within a given year). In models for relative warps (RelW2–RelW6), CentroidSize was included as a covariate to account for allometric variation in body morphology, so that shape and size could be investigated in separate models. Random effects included a “family” term (f), which accounts for the rearing tank environment (micro or V_f_) of each family from fertilization until 12 months after fertilization before they were combined into tanks based on their cross-type. Additional random effects included maternal environment (m or Dam) and paternal environment (s or Sire) effects and the random effect of animal (a).

Animal models for continuous traits (mo12Length, mo12Weight, mo12Kfact, mo15Length, mo15Weight, mo15Kfact, IGRL1, IGRW1, mo24Length, mo24Weight, mo24Kfact, IGRL2, IGRW2, AvgPix, CentroidSize, RelW2, RelW3, RelW4, RelW5, and RelW6) and genetic correlations between them were modeled in a residual maximum likelihood (REML) framework in the software program ASReml v3.0 (Gilmour *et al.* 2009). In this REML framework, fixed effects were selected for inclusion in a univariate or multivariate model based on a Wald F-statistic for the effect. Significant random effects were determined by comparing the log-likelihood of a full model to that of a reduced model without the random effect using a likelihood ratio test (LRT). Significance in the LRT was approximated from a χ^2^ distribution with 1 degree of freedom. To test the hypothesis that the additive genetic variance of each trait was significantly different from zero (V_a_ >0), LRT was performed where the log-likelihood of a full model with random animal effect (*i.e.*, its breeding value) was compared to that of a reduced model without the random animal effect as outlined above. Genetic correlations were determined to be significant by comparing the log-likelihoods of full models with genetic covariance unconstrained to those of a reduced model where the genetic covariance was constrained to be 0.

To properly calculate variance components and covariances with the binary life history traits and correlations with those traits, univariate and multivariate animal models were fitted using a Bayesian Markov chain Monte Carlo (MCMC) approach where the posterior distribution was sampled using a Gibbs sampler as implemented in the R package MCMCglmm (Hadfield 2010). When using a Bayesian approach, the specification of prior probability distributions for model parameters is required, and in this instance we selected priors that were vague to induce little bias in parameter estimation. Priors were selected that equally divided the phenotypic variance among the random effects in the model. Vague priors ensure that posterior distributions reflect mainly the information from the data (Charmantier *et al.* 2011), and that the effect of the prior on the posterior estimate is minimized. Prior selection was validated following guidelines set forth in Wilson *et al.* (2010) and in the course notes for the program MCMCglmm (Hadfield 2010). To properly estimate parameters in binary trait animal models, we applied a logit scale and fixed the value of the residual variance to 1. Models were compared and full models were selected using the deviance information criterion (DIC), the Bayesian equivalent of Akaike’s information criterion (AIC), as outlined in McCarthy and Masters (2005). For each model (univariate or bivariate) the MCMC algorithm was run for at least 100,000 iterations (maximum of 2,000,000) with a burn-in of at least 10,000 iterations (maximum 100,000), and at least 1000 samples (maximum of 2000) were evaluated from the chain. Lag autocorrelations between intervals of accepted models were less than 0.1, and the posterior distributions of all variance components were scrutinized for normality. Examination of the posterior distributions of each model parameter allowed for the evaluation of uncertainty surrounding the estimate. We calculated the regions of 95% highest posterior density (HPD), which provide a conservative measure of uncertainty. As a measure of the central tendency of the estimated parameters we also report the modal value. Binary trait heritability was estimated in a similar manner as defined above; however, an additional weight is applied to the denominator to account for the logit scale that was used for the binary trait distribution, in this case *h^2^* = V_A_/ [V_P_ + (π^2^/3)], as described in the course notes for the program MCMCglmm (Hadfield 2010).

## Results

### Pedigree

In total, 16,139 samples across three generations were used in quantitative genetic analyses, including 73 P_1_ founders, 6593 F_1_, and 9473 F_2_ samples. The F_1_ generation was derived from 42 dams and 31 sires and partitioned into 75 full-sib and half-sib families with an average family size of 88 offspring. The F_2_ generation was derived from 41 dams and 53 sires and partitioned into 69 full-sib and half-sib families with an average of 137 offspring per family. Families were partitioned further by cross-type, with the A×A cross-type broken down into 38 families (3804 offspring) across both generations, A×R broken down into 25 families (2779 offspring) across both generations, R×R broken down into 29 families (2709 offspring) across both generations, R×A broken down into 32 families (3108 offspring) across both generations, AR×AR broken down into 10 families (1800 offspring) in the F_2_ generation, and RA×RA broken down into 10 families (1866 offspring) in the F_2_ generation.

### Phenotypic traits

#### Life history classification:

The overall life history classification of 15,635 rainbow trout at 24 months after fertilization was estimated based on overall body morphology, coloration, and maturity status, whereas 431 individuals could not be definitively assigned to one category based on our criteria and were assigned a missing value for life history. Putative smolts represented the largest life history class, with 71.6% (n = 11,191) of the total juveniles categorized in this group. Indeterminate juveniles represented approximately 4% of the total (n = 614), whereas immature resident parr represented 11.7% (n = 1837) and mature fish represented 12.7% (n = 1993) of the total. The overall average percent of offspring per family classified as smolts at age 24 months after fertilization was 68% (min 2%, max 99%), with the average percent in the F_1_ generation as 60% (min 2%, max 99%) and the average percent in the F_2_ generation as 78% (min 27%, max 98%) (Table 2). The overall average percent of offspring per family that had reached sexual maturity and were thus classified as mature by age 24 months was 13% (min 0%, max 50%), with the average percent in the F_1_ generation as 13% (min 0%, max 50%) and the average percent in the F_2_ generation as 12% (min 0%, max 41%) (Table 2).

**Table 2:** Minimum, average, and maximum proportion of offspring per family

	Proportion of Smolts Per Family				
Group	Minimum	Average	Maximum	nFam	nInd
**A×A**	0.34	0.71	0.98	38	3804
**A×R**	0.22	0.63	0.98	25	2779
**R×R**	0.02	0.56	0.85	29	2709
**R×A**	0.31	0.72	0.99	32	3108
**AR×AR***^a^*	0.63	0.81	0.95	10	1800
**RA×RA***^a^*	0.55	0.83	0.97	10	1866
**F_1_ Generation**	0.02	0.60	0.99	75	6593
**F_2_ Generation**	0.27	0.78	0.98	69	9473
**Global**	0.02	0.68	0.99	144	16,066

	Proportion of Mature Per Family				
Group	Minimum	Average	Maximum	nFam	nInd
**A×A**	0.00	0.10	0.50	38	3804
**A×R**	0.01	0.14	0.41	25	2779
**R×R**	0.00	0.16	0.33	29	2709
**R×A**	0.00	0.12	0.34	32	3108
**AR×AR***^a^*	0.03	0.15	0.31	10	1800
**RA×RA***^a^*	0.01	0.11	0.41	10	1866
**F_1_ Generation**	0.00	0.13	0.50	75	6593
**F_2_ Generation**	0.00	0.12	0.41	69	9473
**Global**	0.00	0.13	0.50	144	16,066

Minimum, average, and maximum proportion of offspring per family classified as putative smolts or sexually mature at age 24 mo after fertilization for the given group (cross type or generation), including the number of families in each group, and the number of individuals classified per group.

a This cross type only occurs in the F_2_ generation.

#### *Body length*, *weight*, *condition factor*, *and growth rates:*

**Figure 3:**
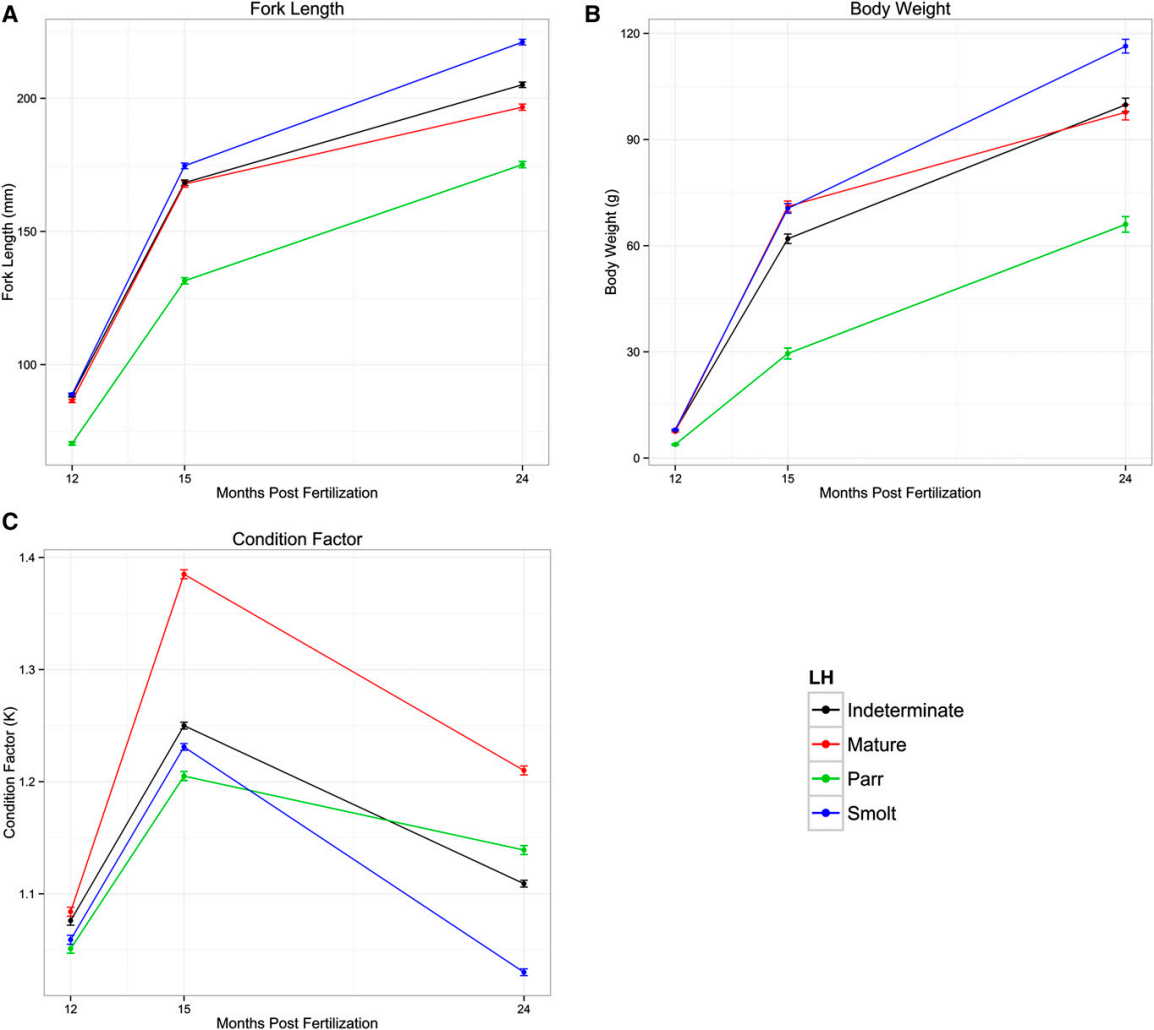
Mean (± SE) of (A) fork length, (B) body weight, and (C) condition factor across the three sampling time points (12, 15, and 24 months after fertilization) for each life history class.

**Figure 4:**
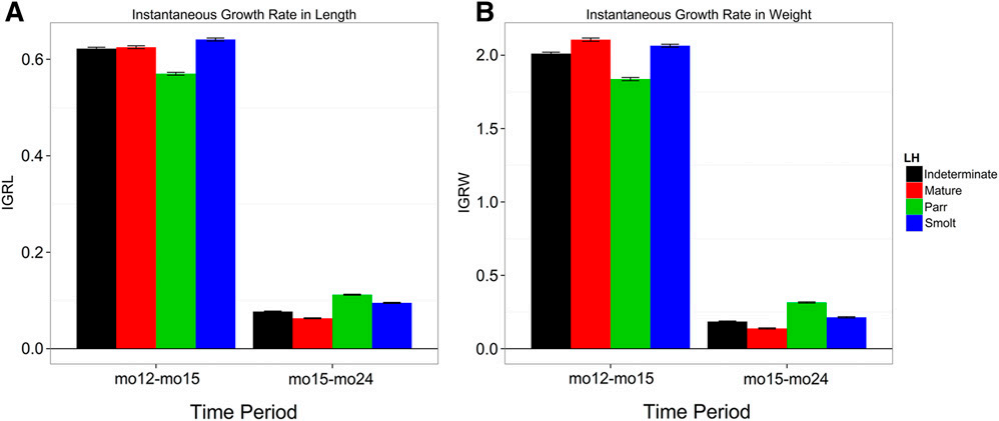
Mean (± SE) of instantaneous growth rates for (A) fork length (IGRL) and (B) body weight (IGRW) across the two sampling time intervals 12 to 15 months (mo12–mo15) and 15 to 24 months (mo15–mo24) after fertilization for each life history class.

Body length, body weight, condition factor, and growth rates in length and weight were measured in 15,571 to 16,050 individuals spanning both F_1_ and F_2_ generations and are summarized in Table 1. ANOVA revealed significant effects of life history classification on all of these traits (*P* < 0.0001), with *t*-tests showing significant differences between the classes as summarized in Figure 3 and Figure 4 and Supporting Information, Table S1. In general, throughout the three sampling time points smolts had the longest body lengths and largest weights, whereas resident parr displayed the shortest lengths and lowest weights (Figure 3, A and B and Table S1). Condition factor, however, was greatest in mature resident fish at all three time points, with resident parr displaying the lowest condition factors at the first two points and smolts displaying the lowest condition factor at the final time point (Figure 3C and Table S1). Differences in mean growth rates were also significant between the life history classes, with smolts having the greatest growth rate in length between 12 and 15 months after fertilization and resident parr having the lowest. During the second interval between 15 and 24 months after fertilization, resident parr had the greatest mean growth rate in length, whereas resident mature had the lowest (Figure 4A). For growth rates in body weight, resident mature fish showed the greatest rate in the first interval, with resident parr showing the lowest rates; conversely, resident parr showed the greatest rates of growth in weight during the second interval with resident mature having the lowest (Figure 4B).

#### Body morphology:

Body morphology was analyzed in a subset of 1985 F_2_ fish, with some individuals being removed from the analysis due to poor-quality digital images. In total, 22 relative warps were generated from the 13 landmarks in addition to the CentroidSize. Life history classification explained a significant proportion of the variation in CentroidSize (*F*_3,1964_ = 65.92, *P* < 0.0001) with mean values significantly different between the life history classes, smolts having the largest mean CentroidSize, and resident parr having the smallest (Table S1).

**Figure 5:**
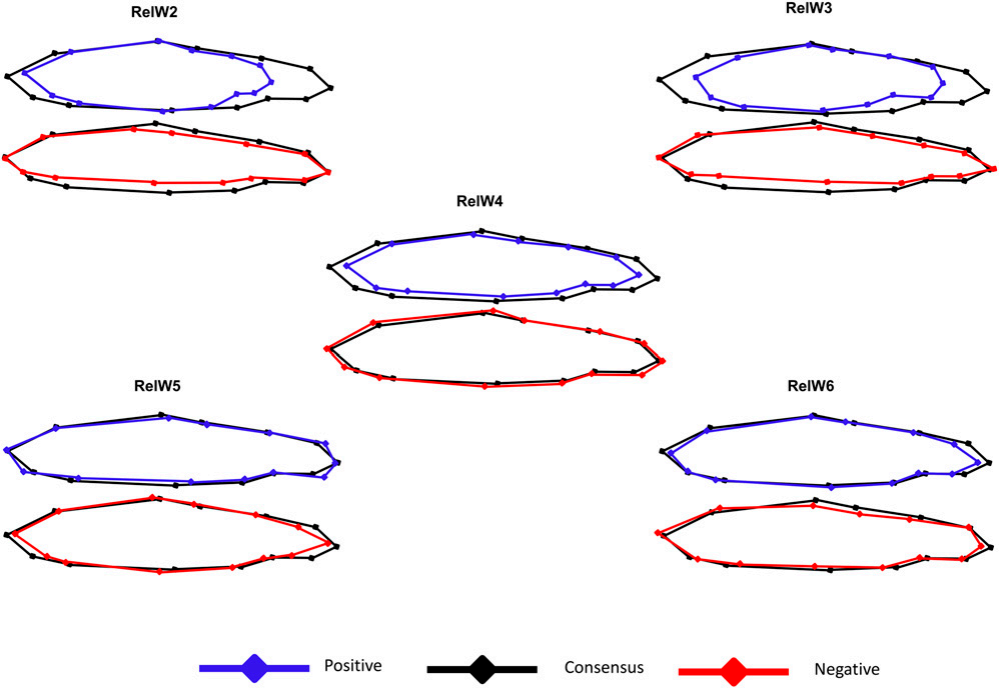
Relative warps from thin plate spline analysis of body morphology. RelW2–RelW6 explain collectively 44% of the variation in body shape. Extreme positive (blue) and negative (red) values of each relative warp are presented in comparison to the consensus shape (black).

Of the 22 relative warps, the first six (RelW1–RelW6) explained 80% of the total variation in body shape and were considered for further analysis. The first relative warp (RelW1) explained the most variation in shape (36.69%) but corresponded to extreme sagging of the caudal fin and peduncle, which is believed to be largely an artifact of anesthesia (see Hecht *et al.* 2012 for details) and has been previously documented in fish (Albert *et al.* 2008). Although RelW1 was found to have a low, albeit statistically supported heritability (h^2^ = 0.03 ± 0.02, *P* = 0.05), it was removed from further analysis and discussion because the shape variation predominantly consisted of what was considered to be an unnatural morphology. The heritability of RelW1 likely reflects true heritable shape variation captured in this warp, but that cannot be dissected from the variation correlated with the bending of the tail. The remaining relative warps 2–6 collectively explained 44% of the total body shape variation, with each explaining 21.06–3.89% of the variation. When comparing the extreme positive and negative values of a warp with the consensus shape, variation in RelW2–RelW6 can largely be described as variation in dorsal-ventral body depth (landmarks 3, 4, and 11), caudal peduncle length and depth (landmarks 4–11), and head morphology (landmarks 1, 2, 11, 12, and 13) (Figure 2). Details regarding the shape variation explained by RelW2–RelW6 can be found in Table 3 and are illustrated in Figure 5.

**Table 3:** Body morphology defined and PVE by each relative warp

Relative Warp	PVE	Body Shape Defined
RelW2	21.06	Length and depth of caudal peduncle and posterior body (landmarks 4–11), dorsal-ventral body depth(landmarks 3, 4, and 11), and head depth (landmarks 2 and 12)
RelW3	8.13	Anterior body length (landmarks 2, 3, 11, and 12), dorsal-ventral body depth (landmarks 3, 4, and 11), and length of the caudal peduncle (landmarks 6-8)
RelW4	5.8	Dorsal-ventral body depth (landmarks 3, 4, and 11), dorsal fin base length (landmarks 3 and 4), snout shape (landmarks 1, 12, and 13), and caudal peduncle length (landmarks 4, 5, and 8–11)
RelW5	5.17	Anterior body length (landmarks 2, 3, 11, and 12), lower snout morphology (landmarks 1, 12, and 13), and caudal peduncle length (landmarks 5–9)
RelW6	3.89	Head morphology (landmarks 1, 2, 11, and 12), caudal peduncle length (landmarks 5–10), and anterior ventral body length (landmarks 10–12)

PVE, percent of the total body shape variation explained.

**Figure 6:**
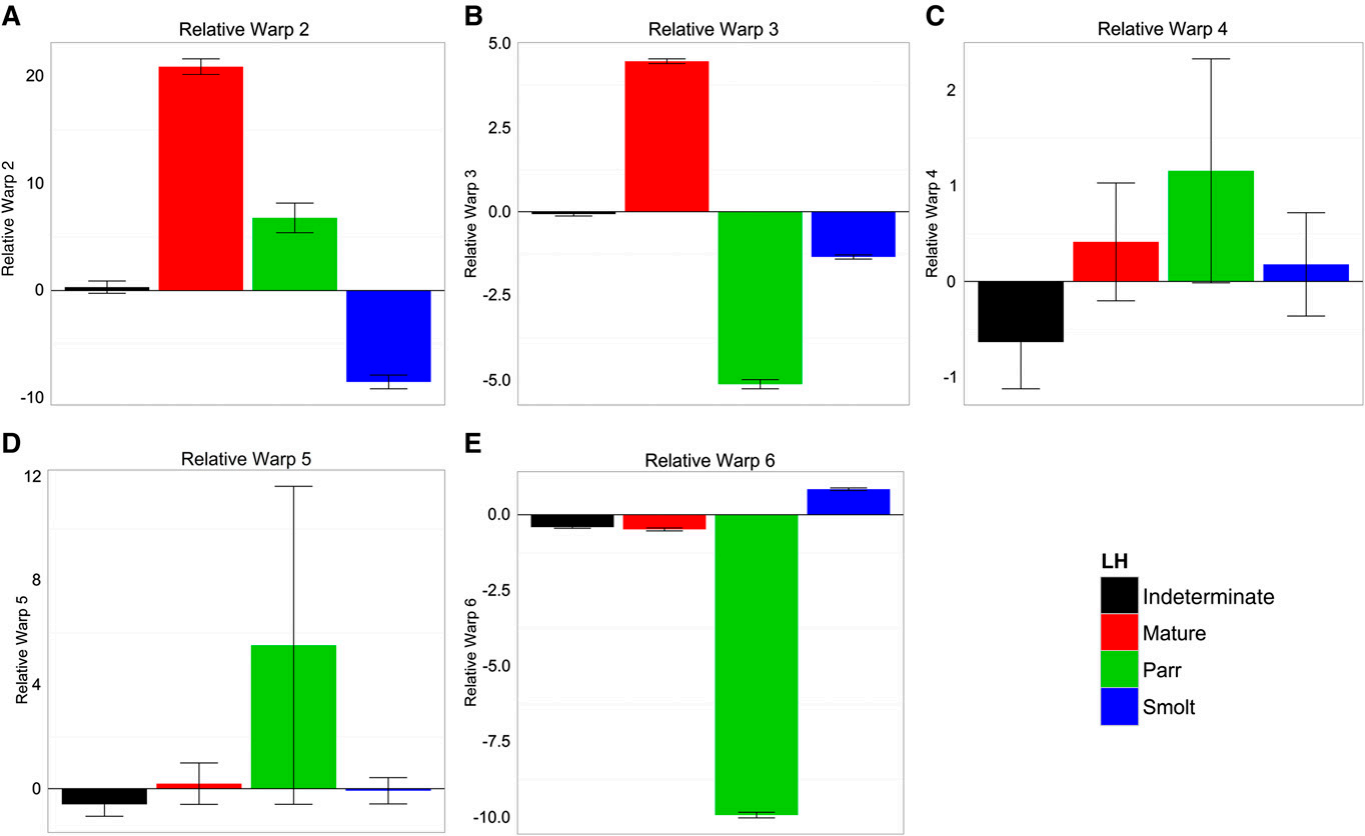
Mean (± SE) of (A) relative warp 2 (RelW2), (B) relative warp 3 (RelW3), (C) relative warp 4 (RelW4), (D) relative warp 5 (RelW5), and (E) relative warp 6 (RelW6) for each life history class measured 24 months after fertilization.

Life history classification explained a significant proportion (*P* < 0.0001) of the variation in RelW2, 3, 5, and 6, but not for RelW4 (Table S1). RelW2 explained 21.1% of the total shape variation and is explained by variation in the length and depth of the caudal peduncle, dorsal-ventral body depth, and head depth (Figure 5 and Table 3). Positive values of RelW2 indicate individuals with deeper but shorter heads and bodies and shorter caudal peduncles, whereas negative values of RelW2 indicate individuals with shallow, elongated heads and bodies, and a longer caudal peduncle. Mature fish, on average, exhibited the most positive mean values of this warp, and smolts exhibited the most negative scores (Figure 6 and Table S1). RelW3 explained 8.1% of total shape variation and encompasses variation in anterior body length, dorsal-ventral body depth, and length of the caudal peduncle (Figure 5 and Table 3). Positive values of RelW3 indicate individuals with shorter anterior body lengths, deeper dorsal-ventral bodies, and shorter but deeper caudal peduncles, whereas positive values of this warp indicate individuals with longer anterior body lengths, shallower dorsal-ventral bodies, and longer and slimmer caudal peduncles. Mature fish had the most positive mean values of this warp, whereas resident parr had the most negative values (Figure 6 and Table S1). RelW4 explained 5.8% of the total shape variation in dorsal-ventral body depth, dorsal fin base length, snout shape, and caudal peduncle length (Figure 5 and Table 3). There was no significant effect of the life history classification on this metric of morphology, and no difference in mean values between the classes (Figure 6 and Table S1). RelW5 explained 5.2% of the total shape variation and includes variation in anterior body length, lower snout morphology, and caudal peduncle length, with the most positive values indicating longer anterior bodies, shorter lower snout morphology, and deeper caudal peduncles, whereas the negative values of this warp indicate shorter anterior bodies, longer lower snout morphology, and shallower and more pointed caudal peduncles (Figure 5 and Table 3). The most positive values of RelW5 were seen in resident parr, whereas the most negative values were found in mature, indeterminate, and smolt classes (Figure 6 and Table S1). RelW6 explained 3.9% of the total shape variation and involves variation in head morphology, caudal peduncle length, and ventral body length in the anterior end. Positive values of this warp indicate shorter head morphology, longer ventral features, and a narrow and pointy caudal peduncle, whereas negative values represent longer head morphology, shorter ventral features, and a broader caudal peduncle (Figure 5 and Table 3). The most positive values of this warp were found among smolts, whereas the most negative values were found in resident parr (Figure 6 and Table S1).

#### Skin reflectance:

**Figure 7:**
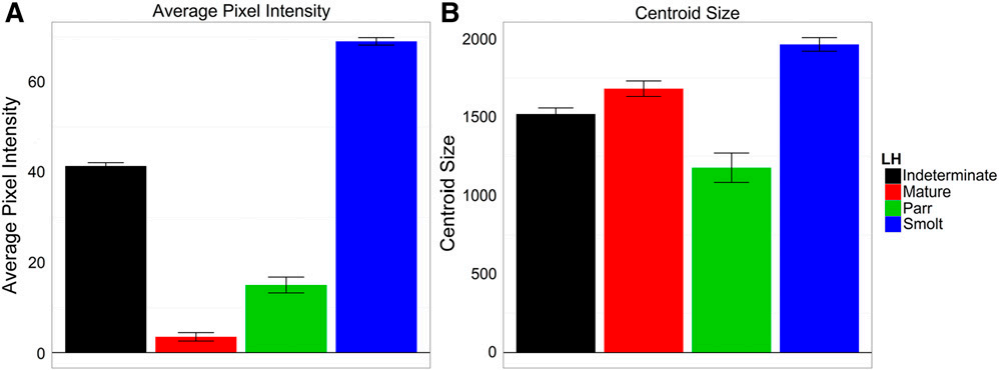
Mean (± SE) of (A) average pixel intensity (AvgPix) and (B) centroid size (CentroidSize) for each life history class measured 24 months after fertilization.

Silvering and reflectance in the skin is one of the most prominent morphological changes that occur in smolts and was measured by quantifying the average white pixel intensity of a defined region on the left lateral side of the body (Figure 2) in 2057 fish. Life history classification explained a significant proportion of the variation in average white pixel intensity, with smolts having on average the most reflective skin and the highest mean white pixel intensity between the life history classes, whereas mature parr had the least reflective skin, with the lowest white pixel intensity (Figure 7 and Table S1).

### Statistical analysis

#### Discriminant function analysis:

The DFA were used to determine the predictive power of the individual phenotypic traits for estimating the life history classification of an individual and were run using three subsets of the data. The first dataset included 15,471 individuals from both F_1_ and F_2_ generations and all of the size and growth-related traits (mo12Length, mo12Weight, mo12Kfact, mo15Length, mo15Weight, mo15Kfact, IGRL1, IGRW1, mo24Length, mo24Weight, mo24Kfact, IGRL2, and IGRW2). This model was able to properly assign 87.0% of individuals to their life history classification. The second model included the same traits as the first, but on a reduced dataset, which only included 8925 F_2_ individuals. This model properly assigned 89.5% of the individuals to their life history class and demonstrates that the ability to assign life history in both generations based on the size and growth-related phenotypes was approximately the same, although the DFA based only on the F_2_ dataset was slightly more accurate. The third model included the size and growth traits from the first two models, but also incorporated all of the morphological traits (AvgPix, CentroidSize, RelW2, RelW3, RelW4, RelW5, and RelW6) and was run on 1882 F_2_ individuals that had complete observations for each of the traits. This model correctly assigned 90.2% of the individuals to their life history class and indicates that the addition of the morphological data did not detectably improve our ability to classify individuals into life history classes (89.5% *vs.* 90.2%) in the F_2._ These results also suggest that the phenotypic traits measured herein can place an individual in its life history classification with a high degree of accuracy, and that a large part of the phenotypic differentiation among the life history types has been captured by measuring these traits.

#### Phenotypic correlations:

**Figure 8:**
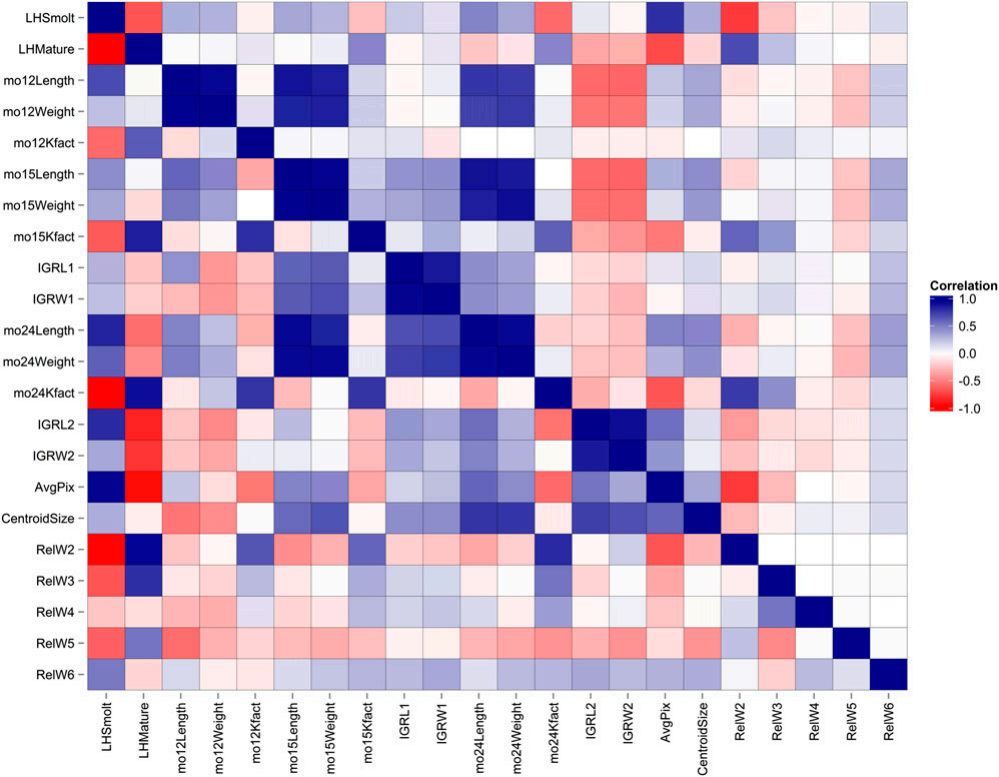
Heatmap representing strength and direction of phenotypic correlations above the diagonal and genetic correlations below the diagonal. Blue cells indicate positive correlations, red cells indicate negative correlations, and white cells represent no correlation. The strength of the correlation is indicated by the saturation of the color within the cell. LHSmolt and LHMature are binary traits, whereas all others are continuous traits (see *Materials and Methods* for details).

Phenotypic correlations were measured between the 20 continuous and the two binary traits. Correlations between these 22 traits ranged from being strongly negative (max −0.76) to strongly positive (max 0.96), with most correlations being moderate in size. In total, 192 significant correlations were detected from 231 off-diagonal correlations (Figure 8 and Table S2). This pattern reflects the complex nature of this migratory syndrome with many correlations between several different size, growth, and morphological related traits. Among the strongest correlations with the LHSmolt binary life history classification was a negative correlation with body condition factor at month 24 (mo24Kfact, r = −0.58, *P* < 0.0001), indicating that smolts were slimmer for their length than non-smolts. Not surprisingly, LHSmolt also had a strong positive correlation with large values of skin reflectance (r = 0.79, *P* < 0.0001), indicating that smolts have more reflective skin with higher white pixel intensity levels than the other classes. Smolts also had a strong negative correlation with RelW2 (r = −0.75, *P* < 0.0001), which explains variation in posterior body length and dorsal-ventral body depth, negative values of which capture more slender fusiform body shapes expected in migratory fish. Phenotypic correlations with LHMature are generally the opposite of those with LHSmolt in sign, which would be expected because the phenotypic traits measured here were intended to capture differences between resident rainbow trout and migratory steelhead smolts.

#### Heritability:

Heritability was measured in 22 traits, including 20 quantitative traits and two binary life history classifications. Significant heritability estimates for size and growth-related traits (mo12Length, mo12Weight, mo12Kfact, mo15Length, mo15Weight, mo15Kfact, IGRL1, IGRW1, mo24Length, mo24Weight, mo24Kfact, IGRL2, and IGRW2) ranged from 0.1 to 0.62, whereas those for morphological traits (AvgPix, RelW2, RelW3, RelW4, RelW5, and RelW6) ranged from 0.25 to 0.52 (Table 4) using the REML framework. Body condition factor at month 24 (mo24Kfact) had the largest heritability (h^2^ = 0.62 ± 0.07, *P* < 0.001) of any of the quantitative traits, whereas body weight at month 12 (mo12Weight) had the lowest significant estimate of heritability (h^2^ = 0.10 ± 0.04, *P* < 0.01). Significant estimates of fixed and random effects varied by phenotype and generally were conditioned on a fixed generation effect, a fixed cross-type effect, and a random effect of micro (where individual families were reared in separate “micros” for 1 yr after fertilization), and some of the relative warps were conditioned on a fixed effect of CentroidSize (Table 4). The only trait to display a maternal environment effect was RelW3 (m^2^ = V_m_/V_P_ = 0.16 ± 0.07, *P* < 0.05), and none of the traits explored here showed evidence of a paternal environmental effect. Cross-type was included as a fixed effect in animal models to condition on the effects of the experimental mating design used to produce the offspring, which might also include environmental variation experienced by both parents and offspring of each cross-type. Including cross-type as a fixed effect may control for some of the additive genetic variation in our models resulting in a downward estimate of heritability, but this was ultimately a more conservative approach than not controlling for the potentially confounding effects. Analyses for a subset of traits suggest a minor increase in heritability for models in which cross-type was purposely excluded from animal models relative to a model in which it was included as a fixed effect (data not shown).

**Table 4:** Variance component and narrow sense heritability estimates using a REML model framework

Trait	N	*Gen*	*xtype*	*CentroidSize*	Micro (Vf)	Dam (Vm)	Vf (SE)	Vm (SE)	Va (SE)	Vr (SE)	m^2^ (SE), *P*	h^2^ (SE), *P*
mo12Length	16,050	x	x		x		7.6 (1.9)		17.87 (6.82)	87.4 (3.6)		0.16 (0.06), <0.01
mo12Weight	16,001	x	x		x		0.54 (0.13)		0.86 (0.39)	7.68 (0.22)		0.1 (0.04), <0.01
mo12Kfact	15,993	x	x		x		0.00076 (0.0002)		0.0016 (0.0005)	0.0066 (0.00027)		0.18 (0.05), <0.001
mo15Length	15,723	x			x		22.47 (6.15)		120.58 (29.99)	297.81 (15.5)		0.27 (0.06), <0.001
mo15Weight	15,702	x			x		34.05 (9.17)		111.52 (35.83)	613.52 (19.43)		0.15 (0.05), <0.001
mo15Kfact	15,692	x	x		x		0.00045 (0.00013)		0.0034 (0.0007)	0.0047 (0.00034)		0.39 (0.07), <0.001
IGRL1	15,708	x	x		x		0.0005 (0.0001)		0.0024 (0.0005)	0.0027 (0.0003)		0.43 (0.07), <0.001
IGRW1	15,641	x	x		x		0.005 (0.001)		0.027 (0.006)	0.027 (0.003)		0.46 (0.08), <0.001
mo24Length	15,642	x			x		32.51 (9.96)		273.26 (58.92)	439.9 (30.08)		0.37 (0.07), <0.001
mo24Weight	15,641	x			x		60.61 (21.14)		423.82 (109.1)	1514.7 (57.82)		0.21 (0.05), <0.001
mo24Kfact	15,634	x	x		x		0.00045 (0.00013)		0.0064 (0.001)	0.0034 (0.00052)		0.62 (0.07), <0.001
IGRL2	15,588	x			x		0.00004 (0.00001)		0.00011 (0.00003)	0.00032 (0.000015)		0.23 (0.06), <0.001
IGRW2	15,571	x	x		x		0.00028 (0.000064)		0.00073 (0.00022)	0.0037 (0.00012)		0.16 (0.05), <0.001
AvgPix	2057		x						232.51 (61.85)	578.65 (38.56)		0.29 (0.07), <0.001
CentroidSize	1985		x						101,890 (31210)	223760 (18544)		0.31 (0.08), <0.001
RelW2	1985		x	x					81.36 (20.28)	120.43 (11.53)		0.4 (0.08), <0.001
RelW3	1985		x			x		9.31 (4.51)	13.91 (8.33)	33.31 (4.41)	0.16 (0.07), <0.05	0.25 (0.14), <0.01
RelW4	1953		x	x					26.95 (6.43)	24.57 (3.48)		0.52 (0.09), <0.001
RelW5	1985		x	x					17.1 (4.43)	30.78 (2.59)		0.36 (0.08), <0.001
RelW6	1985			x					21.83 (5.14)	23.67 (2.84)		0.48 (0.09), <0.001

Variance component (Vf, Micro; Vm, maternal effects; Va, additive genetic effects; Vr, residual variance), proportion of maternal variance (m2), and narrow sense heritability (h2) estimates including their SE are reported from Gaussian trait animal models fit in a REML framework. Significant (α < 0.05) model effects for each trait animal model are indicated with the presence of an "x" in the effect column for the row trait, where fixed effects are indicated with italicized type. Significance (α = 0.05) of the narrow sense heritability (h2) or proportion of maternal variance (m2) estimates are indicated by *P* from a chi-square test (d.f. = 1) between full and reduced animal models including/excluding random animal (Va) or maternal (Vm) effects, respectively.

Heritability estimates for the binary life history classifications in addition to the quantitative traits were conducted using a Bayesian MCMC statistical framework, which provides a more robust technique for analyzing non-Gaussian traits (Hadfield 2010). For both of the binary life history classifications (LHSmolt and LHMature), the best-fit models were run with 1,100,000 iterations of the MCMC chain, a thinning interval of 1000 iterations, and a burn-in of 100,000 iterations. Lag autocorrelation between the intervals was less than 0.1 for both traits, and posterior distributions of all variance components were normally distributed. Additionally, the fixed effect of generation and the random effect of micro were statistically supported in both models, but no support of a cross-type, maternal or paternal environment effect was found (Table 5). The modal value of the heritability with a 95% HPD interval for LHSmolt was 0.606 (0.386–0.766), whereas that of LHMature was 0.51 (0.335–0.72), suggesting a strong genetic basis for both the migratory and precocious mature life history tactics in this population of rainbow and steelhead trout. Heritability estimates for the quantitative traits were derived from bivariate models with the binary life history classifications. Model effects included the fixed effect of cross-type for each trait, generation for all size and growth-related traits, and the random effect of micro for all traits except RelW3. All of the relative warps, except for RelW3, additionally included a CentroidSize fixed effect. No paternal environment effects were identified in any of the Bayesian models, although a maternal environment effect was supported in RelW3 [m^2^ = V_m_/V_p_ = 0.163 (0.078 – 0.28)], which is consistent with the estimates from the REML models (Table 5). The heritability estimates from the Bayesian models are very similar to those from the REML framework, providing additional support and evidence of the robustness of the model parameters selected in both frameworks. Modal values of heritability estimates of the size and growth-related traits ranged from 0.11 to 0.517 and from 0.2 to 0.531 for the morphological traits (Table 5).

**Table 5:** Variance component and narrow sense heritability estimates using the Bayesian MCMC model framework

Trait	n	*Gen*	*Xtype*	*CentroidSize*	Micro (Vf)	Dam (Vm)	Vf (HPD)	Vm (HPD)	Va (HPD)	Vr (HPD)	m^2^ (HPD)	h^2^ (HPD)
LHSmolt*^a^*	15,021	x			x		0.555 (0.219–1.01)		5.35 (2.63–14.99)	1 (1–1)		0.606 (0.386–0.766)
LHMature*^a^*	15,635	x			x		0.305 (0.004–0.6)		4.39 (1.58–9.82)	1 (1–1)		0.51 (0.335–0.72)
mo12Length	15,621	x	x		x		8.24 (4.12–10.96)		23.37 (10.91–36.47)	86.12 (76.56–90.22)		0.197 (0.098–0.31)
mo12Weight	15,576	x	x		x		0.52 (0.28–0.83)		1.38 (0.34–2.45)	7.52 (6.82–7.95)		0.11 (0.04–0.24)
mo12Kfact	15,569	x	x		x		0.0009 (0.0007–0.001)		0.001 (0.0006–0.002)	0.0007 (0.006–0.007)		0.151 (0.074–0.25)
mo15Length	14,968	x	x		x		23.24 (13.28–39.14)		106.58 (56.45–173.64)	310.11 (271.7–330.9)		0.246 (0.22–0.34)
mo15Weight	15,561	x	x		x		35.66 (19.87–60.71)		78.87 (30.55–176.23)	622.15 (578.95–657.92)		0.135 (0.04–0.22)
mo15Kfact	14,943	x	x		x		0.0008 (0.0006–0.001)		0.003 (0.002–0.004)	0.005 (0.004–0.006)		0.378 (0.213–0.467)
IGRL1	15,567	x	x		x		0.0009 (0.0006–0.0012)		0.002 (0.001–0.003)	0.003 (0.002–0.003)		0.357 (0.23–0.50)
IGRW1	14,904	x	x		x		0.008 (0.005–0.011)		0.025 (0.016–0.04)	0.03 (0.022–0.033)		0.39 (0.29–0.58)
mo24Length	15,008	x	x		x		36.85 (22.59–63.09)		272.36 (156.03–368.88)	439.73 (392.64–503.27)		0.366 (0.23–0.47)
mo24Weight	15,620	x	x		x		90.54 (49.24–141.26)		362.7 (160.81–561.79)	1543.56 (1426.14–1647.66)		0.182 (0.09–0.27)
mo24Kfact	15,002	x	x		x		0.0008 (0.0005–0.0013)		0.006 (0.004–0.008)	0.004 (0.003–0.005)		0.517 (0.38–0.71)
IGRL2	14,955	x	x		x		0.00004 (0.00003–0.00006)		0.0001 (0.00007–0.0002)	0.0003 (0.00028–0.00034)		0.235 (0.15–0.37)
IGRW2	14,940	x	x		x		0.0004 (0.0003–0.0005)		0.0007 (0.0004–0.0013)	0.0037 (0.003–0.004)		0.16 (0.10–0.25)
AvgPix	2040		x		x		61.18 (20.99–102.56)		311.84 (126.66–474.12)	464.95 (369.25–558.82)		0.359 (0.17–0.52)
CentroidSize	1702		x		x		153,414.9 (79,715.6–251,570.9)		165,176.5 (47,570.9–384,227.9)	193,389.3 (71,583.0–248,689.4)		0.28 (0.125–0.70)
RelW2	1968		x	x	x		13.57 (5.47–56.8)		80.782 (50.68–124.5)	102.01 (77.15–115.71)		0.4 (0.27–0.58)
RelW3	1968		x			x		8.79 (4.81-21.95)	39.07 (23.4–57.3)	19.99 (9.79–27.81)	0.163 (0.078 - 0.28)	0.531 (0.354–0.753)
RelW4	1968		x	x	x		7.76 (4.51–14.73)		28.29 (14.45–48.83)	24.4 (14.5–31.82)		0.522 (0.26–0.70)
RelW5	1968		x	x	x		4.57 (2.02–12.48)		10.88 (4.81–25.65)	33.81 (26.21–38.0)		0.38 (0.14–0.51)
RelW6	1702		x	x	x		3.96 (1.54–7.45)		8.5 (4.07–16.47)	31.62 (26.10–34.35)		0.2 (0.10–0.36)

Modal values of random variance components (Vf, Micro; Vm, maternal effects; Va, additive genetic effects; Vr, residual variance), proportion of maternal variance (m2), and narrow sense heritability (h2) estimates including their 95% highest posterior density (HPD) intervals are reported from animal models fit in a Bayesian MCMC framework. Supported model effects for each trait animal model are indicated with the presence of an ’x’ in the effect column for the row trait, where fixed effects are indicated with italicized type.

a Variance estimates based on logit scale with residual variance set to 1.

#### Genetic correlations:

Genetic correlations were measured between 22 traits, including 20 continuous traits and two binary life history classifications in two statistical frameworks, REML and Bayesian, respectively. Genetic correlations between the 22 continuous traits ranged from being strongly negative (max −0.99) to strongly positive (max 0.98), with most correlations being moderate in size (Figure 8 and Table S2). In total, 91 significant genetic correlations were detected from 231 off-diagonal correlations, including 64 positive and 27 negative correlations. The binary trait LHSmolt had strong positive genetic correlations (*r*_G_ > 0.5) with the traits mo12Length, mo24Length, mo24Weight, IGRL2, AvgPix, and RelW6, but had strong negative genetic correlations (*r*_G_ < −0.5) with the traits mo12Kfact, mo15Kfact, mo24Kfact, RelW2, RelW3, and RelW5. Conversely, the binary trait LHMature had strong positive genetic correlations (*r*_G_ > 0.5) with the traits mo12Kfact, mo15Kfact, mo24Kfact, RelW2, RelW3, and RelW5, but strong negative genetic correlations (*r*_G_ < −0.5) with the traits mo24Length, IGRL2, IGRW2, and AvgPix. In total, the binary trait LHSmolt had 18 significant genetic correlations, lacking only correlations with the traits mo12Weight, mo15Weight, and RelW3, whereas LHMature had 12 significant genetic correlations. The trait with the fewest significant genetic correlations was mo12Weight, having only genetic correlations with mo12Length and RelW4. Considering all genetic correlations as a whole provides insight into the complexity of this life history, wherein size, growth, and morphological traits are strongly genetically correlated with one another, and with the propensity to migrate. A complete dataset including pedigree and family identification and quantitative traits is provided in Table S3.

## Discussion

Although migration-related traits and the overall migratory life history within salmonid fishes are influenced by environmental factors (Hoar 1976; Folmar and Dickhoff 1980; Wedemeyer *et al.* 1980; Sloat *et al.* 2014), we demonstrate that there is significant heritable variation. Our quantitative genetic analysis of several size, growth, and morphological traits related to migration in juvenile rainbow and steelhead trout illustrate the tremendous genetic variation between freshwater resident and anadromous migratory life history types within the experimental population investigated here. This examination has additionally identified the complex correlated nature of the collection of traits that make up part of the migratory syndrome in this species, suggesting that these migration related traits likely evolve not in isolation, but as a suite of coordinated characters. Furthermore, the phenotypic and genetic correlations between growth and size-related traits at early juvenile life stages and migratory propensity at later life stages support the possibility that one could predict the life history trajectory of an individual based on expressed phenotypic traits during juvenile development in this population. The large heritability estimates identified across the migration-related characters and the binary life history traits measured here suggest that these traits could respond rapidly to selection. Nevertheless, the substantial additive genetic variances identified suggest that selection has maintained variance within the traits that make up part of the migratory syndrome in this population, rather than favoring an optimal life history or phenotype. This could be the result of fluctuating heterogeneous environmental conditions (Spieth 1979), where some environmental inputs or years could favor the anadromous migratory life history whereas others favor the resident life history, resulting in an overall maintenance of genetic and phenotypic variation in this population. It is also possible that frequency-dependent selection might play a role in maintaining genetic variation in life history trajectories, where increased frequency of resident rainbow trout (or steelhead trout) might allow selection to favor migratory steelhead trout (or resident rainbow trout) until the frequencies shifted in time to favor resident rainbow trout (or steelhead trout) again.

Our estimates for the heritability of life history tactic are moderate to high, with a modal estimate of 0.61 (0.386–0.766) for the binary migratory life history (LHSmolt) and 0.51 (0.335–0.72) for precocious maturation (LHMature). Although caution is advised in comparing heritability estimates between species and between wild and experimental populations given that heritability estimates are a component of the population and environment studied (Falconer and Mackay 1996), our results do fall within the range of estimates from previous studies of threshold life history tactics in animals (Roff 1996) and within salmonid fishes (Thériault *et al.* 2007; Carlson and Seamons 2008; Abadía-Cardoso *et al.* 2013). The binary life history trait LHSmolt exhibits a strong negative genetic correlation with LHMature (*r*_G_ = −0.99; 95% HPD interval −0.999 to −0.98), which supports the hypothesis that there is a strongly canalized trade-off during development between early maturation and smoltification (Thorpe 1994; Thorpe and Metcalfe 1998). Given that these life history traits were measured as mutually exclusive binary classifications, this is not a surprising result. However, a strong negative genetic correlation was also found when considering genetic covariance between the proportion of smolts and the proportion of mature offspring produced in an analysis of F_1_ families from this same study population (Thrower *et al.* 2004a). This could have important demographic implications for this population because conditions that favor the migratory life history will necessarily reduce the propensity of precocious maturation; conversely, favorable conditions for early maturation will reduce the propensity of migratory smolts. Our results also support the conclusion of Thrower *et al.* (2004a) that selection against smoltification in this population could lead to a decrease in the age at sexual maturation. This is important within the Sashin Creek system, where rainbow trout from the anadromous portion of Sashin Creek were transplanted above two barrier waterfalls into Sashin Lake to colonize a previously barren habitat (Thrower *et al.* 2004a,b). Anadromous smolts out-migrating from Sashin Lake must pass over two large barrier waterfalls and are not able to return to Sashin Lake to contribute their alleles to the gene pool; thus, there is strong selection against the migratory life history in the lake. The moderate to high estimates of heritability suggest these life history tactics could respond rapidly to selection, but the strong negative genetic correlation suggests evolutionary forces must act within the constraints of this correlation, at least in the near-term. However, although approximately half of the variance in both migratory and precocious mature life histories can be attributed to additive genetic variance, the other half is due to nonadditive genetic (epistasis and dominance) and environmental effects, which remain to influence the adoption of these tactics. Traits that are highly correlated with the life history categories were also significantly heritable. Traits of body size at 12, 15, and 24 months after fertilization exhibited small to moderate heritability in length (h^2^ = 0.16–0.37) and weight (h^2^ = 0.1–0.21), which are similar but lower than the median of a meta-analysis of the same traits across salmonid fishes (Carlson and Seamons 2008). Body length showed strong to moderate positive genetic correlations with the smolt life history at each time point in this study, although body weight only showed a single significant correlation at mo24, the time at which the smolt characteristics are most evident, and when most individuals have made the decision to migrate or stay. Body length and weight showed no significant genetic correlations with the precocious mature life history in the first two time points, although at 2 yr (mo24) both body length and weight showed moderate negative genetic correlations with the precocious mature tactic. Body condition factor, a component of the relationship between body length and weight, quantifies the level of “plumpness” in a fish, and at the three time points had significant heritability estimates of 0.18 to 0.62. Our heritability estimates for body condition at the first two sampling periods are within the range of previously determined heritability estimates in salmonids for body condition, although the estimate at month 24 is higher than the median and upper limit previously reported (Carlson and Seamons 2008). A strong genetic correlation between life history classification and body condition factor at each time point suggests this trait is a substantial contributor to overall life history tactic, with smolts exhibiting smaller body condition and mature fish exhibiting larger condition at each stage. The significant heritability and genetic correlations with life history suggest, however, that body size and condition are important contributors to the overall life history trajectory. Those fish that are larger in length and lower in condition at time intervals after mo12 are genetically more disposed to being migratory, an outcome also observed in brook trout (*Salvelinus fontinalis*), a related salmonid species (Thériault *et al.* 2007), and in a previous analysis of a subset of fish from this population (Thrower *et al.* 2004a). As early as 12 months after fertilization, we see significant segregation in mean body weight and length between smolts and mature resident fish (Table S1), suggesting that the developmental decision to smolt or remain resident may have occurred sometime within the first year of growth in this experimental population, and time points earlier than 12 months after fertilization are likely critical to the development of a life history tactic. It has been shown that high levels of growth within the first year of rearing led to an increase in precocious male maturation in Chinook salmon (Shearer *et al.* 2006), and perhaps here we have missed the stage when mature life history trajectories experience higher levels of growth than smolts.

Much like the traits of body size and condition, growth rates in length and weight also harbor substantial additive genetic variance. Moreover, the positive genetic correlations between growth rates over the course of this study and the smolt life history type, and the negative correlations with growth and the precocious mature life history, suggest not only that smolts experience more rapid growth than mature fish over their second year of development but also that the genetic mechanisms associated with growth in length and weight are key components of life history trajectory. Life history classification at 24 months after fertilization is highly genetically correlated with characters at early time points in development, and we see segregation in size and growth traits between the life history classes at our earliest sampling periods at age 1. This suggests that the proximate genetic mechanisms underlying life history divergence might already be put in motion as early as 12 months after fertilization. This idea was proposed by Thorpe *et al.* (1998), who suggested that growth thresholds act as developmental checkpoints in early life history stages to signal future life history tactics. A breadth of research suggests that juvenile salmonid growth rates are closely linked to life history decisions (Beckman *et al.* 1999; Larsen *et al.* 2006; Sharpe *et al.* 2007), and whereas environmental effects play a key role in regulating growth rates in steelhead trout (Myrick and Cech 2005; Doctor *et al.* 2014), we show, as others have, that genetic variance also contributes substantially to growth and, subsequently, life history trajectories.

Although many of the quantitative genetic parameters estimated here have previously been explored, albeit in a reduced pedigree from the F_1_ generation (Thrower *et al.* 2004a), we add to previous results genetic correlations between morphological features and the general growth, size, and life history traits. We find morphological traits to be highly heritable and strongly genetically correlated to life history classification. Among the morphological traits measured, skin reflectance is a critical component of both marine and freshwater survival in fishes. Light bellies, silver sides, and dark backs help camouflage smolts in a pelagic marine environment from predators in the open ocean, whereas in freshwater streams and rivers dark and colorful morphologies allow resident fish to blend into shallow rocky substrates. Skin reflectance was found to be moderately heritable (h^2^ = 0.29 ± 0.07) but strongly positively correlated (*r*_G_ = 0.956; 0.90–0.98) with the smolt life history, and strongly negatively correlated (*r*_G_ = −0.92; −0.97 to −0.85) with the mature life history, almost to the point of unity. This is not unexpected because the qualitative assignment of fish to individual life history categories is heavily influenced by subjective judgment of body coloration and morphology. In the case of morphology, skin reflectance is so dramatically different between resident and migratory life history types at 24 months after fertilization that this character likely contributes the most to the overall qualitative morphological classification. Given the moderate heritability and the strong genetic correlation with life history tactic, selection on skin reflectance in either the freshwater or the marine environment could consequently cause a response in life history decision.

Variation in body morphology is closely linked to the ecology and life history of fishes (Keeley *et al.* 2005; Morinville and Rasmussen 2008; Albert *et al.* 2008). In this study, we quantified shape variation using geometric morphometric measures, which were important in distilling the quantitative genetic contribution of morphological variance. CentroidSize is moderately heritable (h^2^ = 0.31 ± 0.08) and has a positive genetic correlation with the smolt life history. This trait represents an overall measure of complete body size, and thus it is not surprising that it is also highly genetically correlated with lengths and weights at 24 months after fertilization. Measures of body shape variation captured in relative warps are all found to have moderate levels of heritability (h^2^ = 0.25–0.52) and fall within the range of heritability estimates previously reported for morphometric variance in salmonids (Carlson and Seamons 2008). Each warp also exhibits moderate to high levels of genetic correlation with the smolt life history classification, but vary in sign. RelW2 reveals a high positive genetic correlation with the smolt life history and a strong negative genetic correlation with the mature life history. This warp is the only warp to show significant differences in mean value between all four categorical life history classifications, and it explained the most variation in body shape of those retained for quantitative analysis. This warp captures substantial variation in caudal peduncle length and depth, dorsal-ventral body depth, and head depth, and it ultimately captures the variation between the slender and fusiform shape of putative out-migrating smolts compared to the deeper-bodied mature resident fish. Slender body shapes reduce drag, allowing for sustained long distance swimming as would be experienced during migration events (Webb 1984). The high heritability and strong genetic correlation of this trait to overall life history tactic suggest that this morphology contributes substantially to migratory life history and is capable of responding to selection.

Genetic correlations among migration-related traits within this experimental population could result either from close linkage among genes associated with these traits or from the pleiotropic actions of the same genes influencing multiple traits. Distinguishing between these two scenarios is not possible without further comprehensive quantitative genetic and functional genomic studies. Although the resident population of *O. mykiss* in Sashin Lake was derived from the migratory steelhead in the lower Creek, over time, linkage disequilibrium between loci in the above-barrier and below-barrier populations could have accumulated in these populations, and could influence the degree of genetic correlation in this study. However, prior genetic analyses of migration and migration-related traits have determined that while there is a genome-wide distribution of effects (Hecht *et al.* 2013; Hale *et al.* 2013), there are also some genetic regions harboring QTL for multiple traits (Nichols *et al.* 2008; Hecht *et al.* 2012). Hecht *et al.* (2012) performed a QTL analysis on a single AR×AR family from the F_2_ generation of the current study population (included also in the analysis herein). In this analysis, several genetic regions associated with multiple QTL were identified, although of particular interest were regions on rainbow trout chromosomes Omy12 and Omy14, which had QTL for more than two traits. Additionally, in a population genomic screen of thousands of markers in wild steelhead and rainbow trout from Sashin Creek, Hale *et al.* (2013) also identified loci on Omy12 and Omy14 that contribute to the genetic differentiation between migrants and residents. On chromosome Omy12, the traits LHSmolt, mo15Kfact, mo24Kfact, IGRL2, IGRW2, CentroidSize, and RelW3 all localize to the same regions with overlapping QTL support intervals (Hecht *et al.* 2012). Here, we find strong genetic correlations (−0.5 > *r*_G_ > 0.3) between 13 of the 21 pairwise correlations of those traits. On chromosome Omy14, the traits IGRL1, IGRW1, mo15Kfact, mo24Kfact, RelW3, and RelW4 all localize to the same region with overlapping QTL support intervals. Here, we find support for moderate to strong positive genetic correlations (*r*_G_ = 0.32–0.95) for 5 of the 15 pairwise correlations. Although it is difficult to know whether the genetic correlations and overlapping QTL represent the pleiotropic effect or close linkage of genes (Flint and Mackay 2009; Stearns 2010), the close genetic relationship between many of these traits and the distribution of QTL suggest evolutionary forces acting on a single trait will be constrained, or correlated phenotypic responses to selection would be anticipated. Furthermore, analysis in this extended population provides additional support for the potential pleiotropic nature of QTL on Omy12 and Omy14 found in Hecht *et al.* (2012), and that these regions deserve further inquiry to identify potential candidate genes of the migratory life history within this population (Hecht *et al.* 2014).

Our results indicate substantial genetic variation in the phenotypes we examined, including the propensity to smolt (a threshold indicator of migratory tendency). This threshold trait expressed considerable additive genetic variance as well as some appreciable phenotypic and genetic covariance with size and growth, a result consistent with the conclusions of Thrower *et al.* (2004a) for the same population. The results herein imply a coordinated developmental syndrome involving growth, size, condition, and migratory tendency, as reviewed by Kendall *et al.* (2015). If true, then this syndrome is likely under complex genetic control involving several genetic factors of varying effect on the threshold phenotype (Nichols *et al.* 2008; Hecht *et al.* 2012, 2013; Hale *et al.* 2013). Our results do not provide a test of alternative models for the inheritance of threshold characters such as LHSmolt. One model involves additive genetic variation in the underlying liability to smolt and a fixed physiological or morphological threshold for expression of that trait (Falconer 1965; Satterthwaite *et al.* 2009). Another model involves a largely environmental influence on growth but genetic variation in a developmental switch point for expression of smoltification (*e.g.*, Aubin-Horth *et al.* 2006). On the surface, our results are broadly compatible with either mechanism. However, when considered in light of growing evidence for extensive plasticity of expression of these traits in juvenile rainbow trout with variation in temperature, our results are arguably more consistent with a model for inheritance that involves genetic variation in a developmental switch point that can be expressed along several distinct growth trajectories highly sensitive to temperature or other environmental influences (*e.g.*, Sloat *et al.* 2014). For example, if the substantial plasticity in development among families in each of two distinct temperature environments observed by Doctor *et al.* (2014) for Puget Sound steelhead also holds for the Sashin Creek population, then a norm of reaction influencing smoltification is a plausible mechanism underlying its expression. Smoltification in relatively slow-growing rainbow trout in cold, high-latitude environments may be more sensitive to growth rate during a critical developmental phase than to a particular size, condition, or physiological state. Regardless of the mode of plasticity, however, the expression of alternative migratory and resident life histories could reflect any of several intrinsic mechanisms, including epigenetic variation influencing expression of genes underlying migratory propensity. Although this possibility has not yet been demonstrated for smoltification in rainbow trout, the complex genetic architecture of smoltification in this species and the protracted developmental trajectory it entails provide ample opportunity for epigenetic control of this migratory syndrome during juvenile life history, with important consequences for fitness.
